# Psoriasis and Seborrheic Keratoses: Insights from Biologic Therapy and Skin Imaging

**DOI:** 10.3390/life15030485

**Published:** 2025-03-17

**Authors:** Florin Ciprian Bujoreanu, Diana Sabina Radaschin, Mihail Alexandru Badea, Laura Bujoreanu Bezman, Carmen Pantiș, Carmen Tiutiuca, Liliana Baroiu, Elena Niculeț, Alina Pleșea Condratovici, Alin Laurențiu Tatu

**Affiliations:** 1 Department of Dermatology, “Saint Parascheva” Infectious Disease Clinical Hospital, 800179 Galati, Romania; florin.bujoreanu@gmail.com (F.C.B.); dianaradaschin@yahoo.com (D.S.R.); alin.tatu@ugal.ro (A.L.T.); 2 Faculty of Medicine and Pharmacy, “Dunarea de Jos” University of Galati, 800385 Galati, Romania; carmen.tiutiuca@ugal.ro (C.T.); liliana.baroiu@ugal.ro (L.B.); elena.niculet@ugal.ro (E.N.); alina.plesea@ugal.ro (A.P.C.); 3 Multidisciplinary Integrated Centre of Dermatological Interface Research (MICDIR), “Dunarea de Jos” University of Galati, 800385 Galati, Romania; 4 Department of Dermatology, University of Medicine, Pharmacy, Science and Technology “George Emil Palade” of Targu Mures, 540142 Targu Mures, Romania; badeamihai2011@yahoo.com; 5 Department of Surgical Disciplines, Faculty of Medicine and Pharmacy, University of Oradea, 1 University Street, 410087 Oradea, Romania

**Keywords:** psoriasis, biological therapies, seborrheic keratoses, reflectance confocal microscopy, dermoscopy, skin cancer

## Abstract

Psoriasis is one of the most frequent immune-mediated chronic inflammatory cutaneous disease that exerts a considerable psychological impact, including low self-esteem, stigmatization, and depression. In recent years, biologic therapies have substantially transformed the therapeutic landscape for individuals with moderate-to-severe psoriasis, shifting treatment towards a more targeted and personalized approach. Seborrheic keratoses (SKs) are common benign skin lesions, and their association with psoriasis and biologic therapy remains poorly understood. Our retrospective study evaluated a small cohort of patients with moderate-to-severe psoriasis undergoing biologic therapy at a tertiary dermatology center in Southeastern Europe to evaluate potential correlations with SK development. Smokers had fewer SKs, whereas postmenopausal women and osteoporosis patients had significantly higher SK counts, implicating hormonal influences. PUVA therapy was linked to an increased SK count, whereas UVB and methotrexate treatments had a lesser effect. These findings suggest that biologic therapy and systemic factors may influence SK development, emphasizing the need for further prospective research.

## 1. Introduction

Psoriasis is a chronic, immune-mediated inflammatory cutaneous disease characterized by recurrent, well-demarcated erythematous plaques topped with silvery scales, primarily affecting extensor surfaces such as the scalp, elbows, knees, and lower back. The disease onset typically has two peaks: one during young adulthood and another in middle-aged patients, although cases have been documented across all age groups, emphasizing its multifactorial nature that combines genetic predisposition with environmental triggers [1]. The impact of psoriasis on quality of life (QoL) is profound, extending well beyond its physical symptoms to include significant psychosocial and economic burdens. Studies indicate that individuals with psoriasis experience higher rates of anxiety, depression, and low self-esteem, often related to the stigmatization and social isolation that accompany visible cutaneous lesions [2].

In recent years, biologic therapies have substantially transformed the therapeutic landscape for individuals with moderate-to-severe psoriasis, shifting treatment towards a more targeted and personalized approach. Traditional systemic agents, such as methotrexate and cyclosporine, have been used with success in psoriasis management and demonstrated clinical efficacy. However, these therapies are often limited by considerable side effects, including hepatotoxicity, nephrotoxicity, and myelosuppression, which can restrict their long-term use [3].

By contrast, biologics represent an innovative approach that precisely targets specific immune components central to the inflammatory pathways in psoriasis. These include tumor necrosis factor-alpha (TNF-α) and interleukins such as IL-17 and IL-23, which are critical mediators in the chronic inflammatory cycle driving psoriatic pathology [4].

The specificity of biologics not only enhances clinical efficacy but also substantially reduces systemic toxicity, thus offering a more favorable safety profile [5]. As a result, biologic therapies have been shown to achieve superior clinical outcomes, with significant improvements in skin clearance, reduced disease severity, and enhanced quality of life for patients, marking important progress in psoriasis treatment [6].

Seborrheic keratoses (SKs) are common benign epidermal tumors that commonly develop in middle-aged and elderly individuals, typically presenting as waxy, raised lesions with a “stuck-on” appearance. These lesions can present variations of color from light tan to black and are most frequently localized on the face, chest, back, and extremities, although SKs can arise anywhere on the skin with the exception of one’s soles, palms, and mucosal surfaces [7].

Seborrheic keratoses (SKs) are primarily age-related benign tumors that can be influenced by a variety of factors, including ultraviolet (UV) exposure, genetic predisposition, metabolic dysregulation, and chronic inflammation. UV exposure is a well-documented risk factor, as it can induce changes in the skin that promote the formation of these lesions, particularly in individuals with fair skin [8]. Genetic predisposition plays a significant role in the development of SKs, as mutations in genes such as fibroblast growth factor receptor 3 (FGFR3) and phosphatidylinositol 3-kinase (PIK3CA) have been implicated in their pathogenesis [9,10].

The inflammatory component observed in some SKs, particularly those that are irritated, may involve the accumulation of lymphocytes and histiocytes, indicating an immune response that resembles mechanisms seen in other inflammatory skin conditions, such as psoriasis [11]. Chronic inflammation is another important factor, as it can lead to the upregulation of immune mediators that promote keratinocyte proliferation and survival, further facilitating the development of SKs [11].

Dermoscopy is often the most preferred non-invasive and quick diagnostic method, and most cases exhibit specific dermoscopic characteristic fissures and ridges, hairpin vessels with white halo, comedo-like openings, and milia-like cysts. Clinicians frequently face a challenge with determining the need to remove a seborrheic keratosis and which treatment modality to use when doing so [12]. Treatment is not usually required, although patients often request removal secondary to irritation or pruritus or for cosmetic reasons.

They frequently present with distinguishing features; however, there can be some overlap in morphology with other malignant skin lesions. It is, therefore, important to recognize these features early in order to distinguish these lesions from other skin tumors [12].

Although SKs are typically asymptomatic, they can become irritated or inflamed. This can result in patients seeking treatment for cosmetic reasons or to rule out malignancy, as SKs can mimic more serious skin conditions, such as melanoma, non-melanoma skin cancer (squamous cell carcinoma and basal cell carcinoma), and other various skin tumors [13]. This connection is particularly relevant, as psoriasis is characterized by dysregulated immune responses leading to keratinocyte hyperproliferation.

On the other hand, melanoma may mimic seborrheic keratoses, leading to incorrect patient management [14]. Overall, seborrheic keratoses are a benign condition, but their clinical presentation requires careful assessment to differentiate them from malignant lesions. In certain cases, non-invasive imaging techniques, such as reflectance confocal microscopy (RCM), can provide valuable diagnostic information, aiding in the differentiation between benign and malignant skin lesions. By offering real-time, high-resolution visualization of cellular structures, RCM has the potential to enhance diagnostic accuracy and support clinical decision making in complex dermatological cases [15].

If there is uncertainty with the diagnosis or other concerns for malignancy, such as ulcerated lesions, a rapid change in size, or overall large, atypical lesions, a skin biopsy would be recommended for confirmation [16].

While these therapies effectively control psoriatic inflammation, their broader impact on other cutaneous structures, including benign proliferative lesions like seborrheic keratoses (SKs), remains poorly understood. Given the immunoregulatory effects of biologic therapies, there is a plausible but underexplored connection between these treatments and the emergence or progression of SKs in psoriasis patients. However, the current literature lacks systematic investigation into this relationship. This study seeks to determine whether biologic therapies influence the development and prevalence of SKs in psoriasis patients and whether different biologic agents (TNF-α inhibitors, IL-17 inhibitors, and IL-23 inhibitors) have distinct effects on SK occurrence. Additionally, the relationship between psoriasis severity, as reflected by PASI scores, and the evolution of SKs during treatment has not been systematically evaluated. Also, we aim to explore the role of patient-specific factors, including age, gender, comorbidities, and prior systemic treatments, in modifying this potential relationship by integrating clinical and dermatological assessment with imaging techniques such as digital dermoscopy or reflectance confocal microscopy.

## 2. Materials and Methods

Design: The current study was conducted as a retrospective observational study, which was carried out in the Dermatology Department of “Sf. Parascheva” Clinical Infectious Diseases Hospital of Galati, Romania, and included 60 patients with moderate-to-severe forms of psoriasis vulgaris undergoing biological therapies over a 5-year period (2019–2024).

Clinical, demographical, and treatment-related data were extracted from medical records, while the photographing and documentation of cutaneous lesions in the study participants was conducted using digital dermoscopy. The extent of the cutaneous lesions and their evolution during treatment were assessed using the Psoriasis Area and Severity Index score (PASI) and the Dermatology Life Quality Index (DLQI).

The inclusion criteria were as follows:

- Adults (≥18 years old) with a confirmed diagnosis of moderate-to-severe-plaque psoriasis, based on clinical and histopathological criteria;
- Patients eligible for biologic therapy according to national treatment guidelines, which includes patients with extensive severity scores (PASI > 10 and DLQI > 10) and those with intolerance, inefficacy, or contraindications to conventional systemic therapies (methotrexate, cyclosporine, acitretin, or UVB therapy);
- Patients undergoing biologic therapy with TNF-α inhibitors, IL-17 inhibitors, or IL-23 inhibitors for at least 12 months;
- The availability of complete medical records, including detailed dermatologic evaluations;
- Patients who agreed to participate after being informed about the benefits of regular dermatoscopic evaluation, including screening and regular monitoring of pigmented skin structures.

Exclusion criteria:

The exclusion criteria for this study included patients with insufficient medical records, those who discontinued biologic therapy, and individuals who were initially eligible but no longer met the national protocol criteria for biologic treatment. Additionally, patients with absolute contraindications to biologic therapy, those receiving other systemic therapies apart from biologics (cyclosporine and methotrexate), and individuals with milder forms of psoriasis (PASI score < 10 and DLQI < 10) typically managed with topical therapies or phototherapy were also excluded.

Data collection and variables:

Patient data were retrieved from electronic and physical medical records, as well as from dermoscopic imaging databases, and the following variables were analyzed:

Demographics and lifestyle factors: age, sex, BMI, nutritional status, environmental background, smoking and alcohol consumption histories, chronic UV exposure history, and phototype classification.

Psoriasis characteristics and severity: clinical manifestations, including psoriatic arthropathy and its distribution (axial/peripheral), as well as genital, palmoplantar, and nail involvement; psoriasis severity was assessed using PASI (Psoriasis Area and Severity Index) scores at baseline, 3, 6, and 12 months, along with DLQI (Dermatology Life Quality Index) scores recorded throughout follow-up.

Biologic therapy and other treatments: The study included an analysis of current and past biologic therapies, dividing patients from the group based on exposure to TNF-alpha inhibitors, IL-23 inhibitors, and IL-17 inhibitors. Additionally, prior use of methotrexate, PUVA therapy, UVB therapy, NSAIDs, chemoprophylaxis with isoniazid for tuberculosis prevention, and beta-blockers was documented.

Comorbidities: The presence of relevant systemic comorbidities was documented, including menopausal status; osteoporosis; cardiovascular diseases (hypertension and ischemic heart disease); hepatic, respiratory, and renal diseases; endocrine disorders (diabetes, thyroid dysfunction); and a history of surgical procedures. Other factors assessed included history of sunburn and the presence of bacterial, viral, or fungal skin infections.

Dermoscopy and skin lesion examination: The total number of seborrheic keratoses, including both pre-existing and newly developed lesions, was recorded. Additionally, other dermatologic findings, including total nevi count, actinic keratoses, lentigo, keratoacanthoma, skin tags, and non-melanoma skin cancers (NMSCs), were assessed through dermoscopic and clinical evaluations.

Statistical analysis: The selected patient data were introduced in an Excel table and then imported and processed with the help of SPSS 29.0. The data were sorted into categories, and then the frequency distribution was carried out. The possible influences between the data were investigated with the help of the Fisher and Chi-squared tests. Descriptive statistics were used for the quantitative variables, and the possible differences were investigated using parametric and non-parametric tests. The following tests for sample comparison were used: the Kolmogorov–Smirnov test, the t-Student test, the ANOVA test, the Mann–Whitney test, and the Kruskal–Wallis test. To investigate the association between two quantitative variables (e.g., the influence of age, the duration of biologic therapy, disease duration, and initial PASI and DLQI scores on the number of melanocytic nevi initially captured, the number of seborrheic keratoses and other cutaneous structures), we calculated Pearson’s correlation coefficient (r), together with its significance level and the corresponding 95% confidence interval, which allowed us to characterize the direction and intensity of this association. While effect size calculations were considered, they were not included due to the retrospective nature of the study and sample size limitations. However, to ensure statistical robustness, multiple comparison adjustments and confidence intervals were applied where necessary. Statistically significant values were those of *p* < 0.05, while values of *p* < 0.01 were highly significant statistically.

Ethical considerations: This study was conducted in accordance with the Declaration of Helsinki and received ethical approval from the Ethical Committee of “Saint Parascheva” Clinical Hospital of Infectious Diseases, Galati, Romania (Approval No. 2/4, dated 26 March 2024), the Ethical Committee of the Medical College of Galati (Approval No. 160, dated 15 February 2024), and the University Ethics Committee of “Dunărea de Jos” University of Galati (Approval No. 14, dated 30 May 2024).

All patients included in the study have given their informed consent. All patient data were fully anonymized to ensure confidentiality.

## 3. Results

### 3.1. Clinical Data

The median duration of biologic therapy in the study cohort was 62.38 ± 6.013 months, with the range spanning from 10 to 180 months (Table 1).

**Figure 1:**
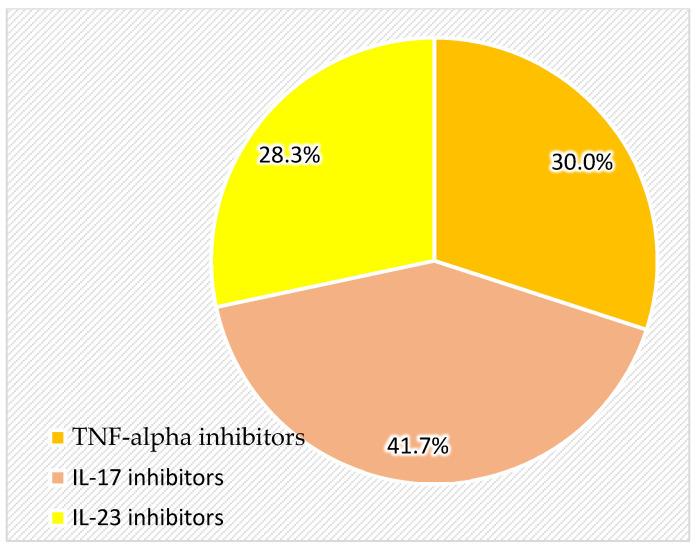
Therapeutic classes of biologics in the study group.

Three types of biologic agents were administered: IL-17 inhibitors, which were the most frequently prescribed (41.7% of patients), followed by TNF-alpha inhibitors (30.0%) and IL-23 inhibitors (28.3%) (Table 2, Figure 1).

The patients’ disease duration varied between 4 and 46 years, with an average of 24.62 ± 1.568 years (Table 3).

The mean age of the patients was 55.42 ± 11.601 years, with a range between 23 and 80 years. There were no statistically significant differences between genders, although women were, on average, approximately 2 years older than men.

At baseline, the mean PASI score was 26.24 ± 1.37, significantly decreasing by 51.6% to 12.71 ± 0.98 at three months, 77.8% to 5.81 ± 0.57 at six months, and 91.3% to 2.28 ± 0.31 at twelve months (*p* < 0.001 for all comparisons). A parallel decline in DLQI scores was documented, with a decrease from 21.07 ± 0.81 at baseline to 2.00 ± 0.39 at 12 months, signifying a 90.5% enhancement in terms of quality of life (Table 4).

These observations suggest a considerable and stable response to biologic therapy, with a substantial proportion of patients reaching a PASI 90 score or nearly complete disease clearance by the one-year point. The observed improvements align with those reported in clinical trials, reconfirming the real-world efficacy of biologics.

**Figure 2:**
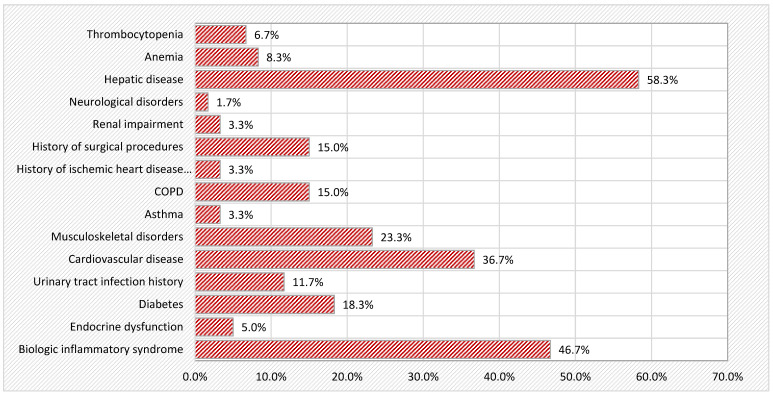
Schematic representation of comorbidities in the study cohort.

The table below provides a summary of the main comorbidities observed in the studied patient cohort, offering an overview of their distribution (Table 5, Figure 2).

### 3.2. Seborrheic Keratoses Findings in Our Study Group

Prevalence and distribution:

The number or seborrheic keratoses ranged from 0 to 23, with a mean of 6.13 ± 0.525. Males and rural patients had slightly higher counts than females and urban patients, although the differences were not significant (Table 6).

Lifestyle and clinical factors:

Smokers had significantly fewer seborrheic keratoses than non-smokers (5.23 ± 4.033 vs. 6.82 ± 4.011; *p* < 0.05).

Postmenopausal women and patients with osteoporosis had significantly higher seborrheic keratosis counts (6.31 ± 2.676 and 9.63 ± 5.680, respectively; *p* < 0.01). Patients with a history of keratoacanthoma exhibited a threefold increase in the number of seborrheic keratoses compared to those without keratoacanthoma history (15.33 ± 8.021 vs. 5.65 ± 3.215; *p* < 0.001) (Table 7).

Biologic therapy and medications:

Treatment with TNF-alpha inhibitors was associated with the highest mean number of seborrheic keratoses (8.50 ± 4.866), significantly greater than that observed in patients receiving IL-23 inhibitors (4.41 ± 2.830; *p* < 0.05).

Patients with a history of using beta-blockers or NSAIDs exhibited higher seborrheic keratosis counts. In contrast, patients with a family history of psoriasis exhibited significantly lower seborrheic keratosis counts compared to those without familial association (4.40 ± 3.112 vs. 6.71 ± 4.208; *p* < 0.05) (Table 7).

Comorbidities and lesion involvement:

Seborrheic keratosis counts were slightly reduced in patients with psoriatic arthropathy, genital involvement, or palmoplantar and nail lesions; however, these differences were marginally significant.

Higher mean counts of seborrheic keratoses were observed in patients who underwent chemoprophylaxis with isoniazid for tuberculosis prevention (and prior PUVA therapy). Lower counts were noted in those with previous MTX treatment or UVB therapy.

**Figure 3:**
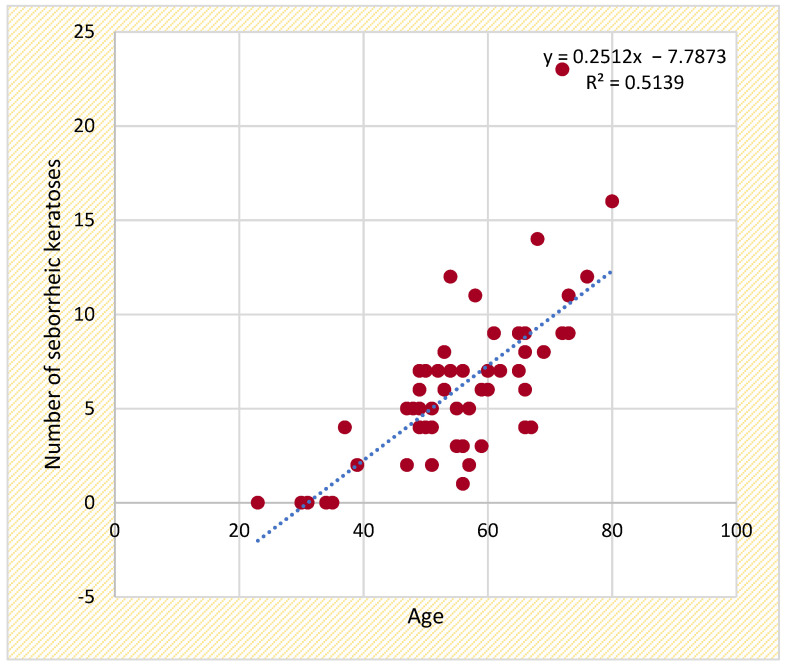
Correlation between patient age and the number of seborrheic keratoses.

Correlations:

The presence of seborrheic keratoses demonstrated a strong positive correlation with age (r = 0.717; *p* < 0.001) (Figure 3) and a moderate correlation with the duration of biologic therapy (r = 0.270; *p* < 0.05). Other factors, such as the BMI, disease duration, and PASI scores, showed minimal influence (Table 8).

In Figure 3, using linear regression analysis, this scatter plot highlights a positive correlation between age and seborrheic keratosis count, suggesting an age-related increase in lesion prevalence.

This scatter plot illustrates the positive correlation between patient age and the number of existing seborrheic keratoses. The trend line equation (y = 0.2512x − 7.7873) suggests that the number of seborrheic keratoses increases with age.

## 4. Discussion

Seborrheic keratoses (SK) are benign epidermal tumors that frequently appear in adults, often being associated with aging and certain dermatological conditions. The link between seborrheic keratoses and biologic therapies, especially in psoriasis patients, has not yet been thoroughly studied, leaving gaps in our understanding of whether these innovative therapies could influence the development of these lesions. Biological therapies, particularly those targeting immune pathways, can lead to changes in the evolution of cutaneous lesions. For instance, the use of anti-PD-1 therapies has been associated with inflammatory responses in pre-existing seborrheic keratoses, suggesting that these treatments can provoke or exacerbate existing skin conditions [17].

This is particularly relevant in patients with psoriasis, who may already have a predisposition to various skin lesions due to their underlying inflammatory condition and the immunological changes induced by this innovative therapy [18,19]. Moreover, specific case reports have documented the emergence of eruptive seborrheic keratoses following the initiation of treatments such as adalimumab, a tumor necrosis factor (TNF) inhibitor commonly used in psoriasis management. In these cases, seborrheic keratoses appeared either concurrently with or following the treatment, indicating a potential link between the therapy and the development of these lesions. The resolution of seborrheic keratoses often correlated with the management of the underlying psoriatic condition, suggesting that the biological therapy may alter the skin’s response to various factors, including the development of seborrheic keratoses [19].

Furthermore, the histopathological similarities between seborrheic keratoses and other epithelial proliferations, such as those induced by chronic viral infections, highlight the complex interaction between immune modulation and skin lesion development. Chronic viral infections can lead to T-cell tolerance, and the restoration of T-cell responses through biological therapies may result in the emergence of previously dormant skin lesions, including seborrheic keratoses. This phenomenon could be particularly relevant in the context of psoriatic disease, where immune dysregulation plays a central role, as biological therapies directly target immune pathways [17].

Beyond the direct effects of biological therapies on seborrheic keratoses, recent research suggests that the inflammatory environment associated with psoriasis and its treatments may also influence overall skin health. Systemic therapies, in particular, have the potential to alter the skin’s barrier integrity and immune response, which could contribute to the development of new cutaneous lesions [20].

The finding that smokers exhibit significantly fewer seborrheic keratoses compared to non-smokers (5.23 ± 4.033 vs. 6.82 ± 4.011; *p* < 0.05) is noteworthy and highlights the intricate relationship between smoking and skin health. Nicotine, a primary component of tobacco smoke, has been shown to express complex effects on keratinocyte behavior. Particularly, nicotine could enhance keratinocyte adhesion, differentiation, and even apoptosis while inhibiting migration. All these mechanisms could potentially suppress the formation of hyperproliferative lesions, such as SKs [21]. This observation may reflect the impact of smoking on the immune microenvironment or keratinocyte behavior, potentially influencing the pathogenesis of seborrheic keratoses.

Additionally, nicotine has been reported to modulate immune responses, exhibiting anti-inflammatory properties in various contexts. This immunosuppressive effect alone might contribute to a reduction in benign epidermal proliferations [22]. However, it is essential to reconsider that smoking is a well-known risk factor for numerous skin conditions, including cutaneous malignancies and delayed wound healing. Consequently, this hypothesis should be interpreted with caution, with further research needed to elucidate the underlying mechanisms. This observation may reflect the impact of smoking on the immune microenvironment or keratinocyte behavior, potentially influencing the pathogenesis of seborrheic keratoses.

Furthermore, the increased prevalence of seborrheic keratoses in postmenopausal women and patients with osteoporosis (6.31 ± 2.676 and 9.63 ± 5.680, respectively; *p* < 0.01) suggests a possible role of bone health and hormonal fluctuations in the development of these lesions. Declining estrogen levels during menopause may impair skin elasticity, repair mechanisms, and immune function, thereby contributing to this trend [19,23].

For example, a study by Saadeh et al. found that postmenopausal women with osteoporosis exhibited a higher prevalence of certain skin conditions, suggesting that the underlying mechanisms of bone health may also affect skin integrity. The hormonal fluctuations that occur during menopause, particularly the decline in estrogen levels, have been implicated in both osteoporosis and the development of SKs. Estrogen is known to play a crucial role in maintaining skin elasticity and hydration, and its deficiency can lead to various skin changes, including the formation of SKs [24].

Furthermore, we present a case report from the literature that highlights a potential connection between hormonal changes and the development of seborrheic keratoses. A 64-year-old postmenopausal women experienced a sudden eruption consisting of multiple seborrheic keratoses after the discontinuation of a long-term estrogen patch. A plausible explanation for this correlation may lie in the protective role of estrogen on skin health. Estrogen is known to regulate keratinocyte proliferation and maintain epidermal homeostasis; hence, its decline during menopause can create a permissive environment for the development of various epidermal proliferations, such as, in our case, multiple seborrheic keratoses. The sudden withdrawal of exogenous estrogen, as seen in this particular case, might worsen the tendency by triggering changes in the activity of keratinocytes or in the skin barrier function. Moreover, the inflammatory environment associated with psoriasis, as in this reported case, may interact with existing hormonal changes and further promote the proliferation of seborrheic keratoses [25]. This observation aligns with our findings that postmenopausal women in our cohort exhibited higher counts of seborrheic keratoses and highlights the role of hormonal changes and inflammation in the development of seborrheic keratoses in postmenopausal women.

The threefold increase in seborrheic keratoses among patients with a history of keratoacanthoma (15.33 ± 8.021 vs. 5.65 ± 3.215; *p* < 0.001) further emphasizes the need to consider a patient’s dermatological history when assessing the risk of developing seborrheic keratoses. This finding is consistent with the literature suggesting that patients with a history of certain skin lesions may be predisposed to developing additional keratotic lesions [26]. However, the specific claim of a threefold increase lacks direct support from the cited studies. In conclusion, while our observation within this cohort suggests a possible association between keratoacanthoma history and the high number of seborrheic keratoses, including a possible threefold increase, this finding is not fully supported by the existing literature. Further research is required to validate these findings and to explore their practical clinical implications for patient monitoring and clinical management.

Furthermore, Botelho et al. discussed the association of seborrheic keratoses with various malignant lesions, including keratoacanthomas. However, the authors did not provide evidence that this association significantly increases the risk of developing seborrheic keratoses. Their findings focused on the prevalence of SKs alongside other skin neoplasms without establishing a direct link [27].

The finding that TNF-alpha inhibitors are linked to a higher average number of seborrheic keratoses (8.50 ± 4.866) compared to IL-23 inhibitors (4.41 ± 2.830; *p* < 0.05) is intriguing and raises questions about how different biologic therapies influence skin health. It is possible that TNF-alpha inhibitors cause immune changes that promote keratinocyte proliferation, making patients more prone to developing these lesions. On the other hand, IL-23 inhibitors, which target a different pathway, seem to have less impact on the development of seborrheic keratoses [28].

TNF-alpha inhibitors can cause a variety of cutaneous reactions, such as the development of new lesions or the exacerbation of pre-existing SKs, according to reports like Sand and Thomsen’s retrospective study [29]. TNF-alpha inhibitors could influence immune regulation in a way that promotes keratinocyte proliferation and SK development. This theory is supported by Eastman et al., who reported cases of eruptive SKs following adalimumab treatment, reinforcing a possible connection [19].

The connection between TNF-alpha inhibitors and the increased incidence of seborrheic keratoses (SKs) raises important questions about the vast dermatologic effects of these biologic therapies in psoriasis patients. TNF-alpha inhibitors, such as adalimumab, etanercept, infliximab, certolizumab pegol, and golimumab, are widely used to manage inflammatory diseases, yet they have been associated with unexpected skin reactions, including the emergence of new lesions or changes in pre-existing conditions [29].

While the exact mechanisms remain unclear, it is likely that TNF-alpha inhibition alters immune regulation and keratinocyte proliferation in ways that promote the development of benign epidermal lesions such as SKs. TNF-alpha plays a central role in immune response regulation, inflammation, and apoptosis. Blocking this pathway can disrupt normal skin homeostasis, potentially leading to increased susceptibility to epidermal proliferations. Some studies have reported that patients taking TNF-alpha inhibitors have a higher risk of developing non-melanoma skin cancers, suggesting that long-term immune modulation may contribute to uncontrolled keratinocyte growth [30]. Although SKs are benign, their increased presence in biologic-treated patients suggests that TNF-alpha blockade might facilitate either the appearance of previously undetectable lesions or the formation of new ones. Additionally, TNF-alpha inhibition is known to affect signaling pathways such as epidermal growth factor receptor (EGFR) and vascular endothelial growth factor (VEGF), both of which are key regulators of keratinocyte proliferation and differentiation [29].

Beyond immune modulation, environmental factors such as UV exposure may also play a role in this increased incidence of SKs. Immunosuppressive treatments can alter the skin’s ability to handle environmental stressors, potentially making biologic-treated patients more susceptible to UV-related epidermal changes). Furthermore, chronic inflammatory conditions like psoriasis inherently impact skin barrier function, which may further predispose patients to epidermal alterations [31].

Further case reports from the literature support this connection, with Vestergaard et al. documenting the development of SKs in areas of treated psoriasis plaques during efalizumab treatment, an anti-CD11a biologic. The appearance of SKs in these cases highlights the potential role of immune modulation in the pathogenesis of SKs, possibly through altered keratinocyte signaling or inflammatory mediators [32]. In contrast, other biologic therapies, such as PD-1 inhibitors, have been associated with distinct dermatological responses. For example, Rambhia et al. reported a case in which the PD-1 inhibitor nivolumab caused inflammatory changes of seborrheic keratoses, highlighting a mechanism of action that appears to be unrelated to the increased frequency of seborrheic keratoses observed with TNF-alpha inhibitors [17].

Wolner et al. highlighted that treatments like pembrolizumab can lead to changes in pigmentation and other skin lesions, but these effects are not directly comparable to the reactions observed with TNF-alpha inhibitors [33]. Moreover, the pathophysiological mechanisms underlying the effects of TNF-alpha inhibitors on seborrheic keratoses may involve multiple inter-related factors, alterations in cytokine production, and keratinocyte behavior. Burkhart hypothesized that seborrheic keratoses might possess a high irritant threshold, potentially interacting with the profile of cytokines modulated by TNF-alpha inhibitors [34]. This suggests that TNF-alpha, an important mediator of keratinocyte regulation, may influence keratinocyte differentiation and proliferation. Its inhibition could disrupt the balance of cytokines such as IL-1, IL-6, and IL-8, which are key players in skin homeostasis, thereby creating conditions that could lead to the development of seborrheic keratoses [34]. Additionally, TNF-alpha inhibitors might influence the local inflammatory microenvironment by suppressing TNF-alpha-driven signaling while enhancing the activity of other cytokines, such as IL-10 or TGF-beta. This shift in immune signaling could generate an environment favorable for benign epithelial proliferation, contributing to an increase in seborrheic keratoses [35].

In addition, the moderate correlation with the duration of biologic therapy (r = 0.270; *p* < 0.05) suggests that prolonged immune modulation may contribute to seborrheic keratoses development. In the present cohort, the higher counts of SKs observed during the follow-up period appear to be influenced by the extended use over the time of TNF-alpha inhibitors in comparison to IL-23 and IL-17 inhibitors. As the oldest class of biologics available in our country, TNF-alpha inhibitors have been used for significantly longer periods, potentially leading to cumulative effects on keratinocyte dynamics and the formation of other cutaneous structures. Our findings indicate that age, the duration of biologic therapy, and immune modulation may play a role in SK development, highlighting the need for further research into these mechanisms [36].

In comparison, the effects of IL-23 and IL-17 inhibitors on seborrheic keratoses have not been as extensively studied. Mastorino et al. compared the efficacy of anti-IL-17 and anti-IL-23 inhibitors in psoriatic patients but did not report on the incidence of seborrheic keratoses [37]. To the best of our knowledge, no other studies in the literature have specifically examined the impact of IL-23 and IL-17 inhibitors on the development of seborrheic keratoses, highlighting a gap in the current understanding of these therapies’ dermatological effects. This could suggest that the increased frequency of seborrheic keratoses in patients receiving TNF-alpha inhibitors, as opposed to IL-23 and IL-17 inhibitors, may be attributable to the interplay of keratinocyte sensitivity, localized changes in the inflammatory microenvironment, and the prolonged duration of therapy. These findings highlight the need to better understand how TNF-alpha inhibitors and other molecules modulate skin lesion development, especially with their long-term use.

The concept of “psoriasiform keratosis”, as proposed by Walsh et al., is intriguing, as it describes lesions that exhibit characteristics of both seborrheic keratoses and psoriasis. These similarities include irregular verrucous acanthosis, areas of parakeratosis, and neutrophilic infiltrates. This hybrid pathology challenges the traditional demarcation of these two conditions and suggests a deeper mechanistic overlap. The presence of such lesions may indicate overlapping molecular pathways involving keratinocyte proliferation and chronic inflammation, offering deeper insights into the effects of immune modulation on epidermal homeostasis [38].

The association between the higher counts of seborrheic keratoses (SKs) and the utilization of non-steroidal anti-inflammatory drugs (NSAIDs) or beta-blockers constitutes a subject of interest. While specific studies directly correlating these medications to increased counts of SKs are limited, there are indications that certain pharmacological agents may influence skin homeostasis and the development of keratotic lesions. Firstly, non-steroidal anti-inflammatory drugs (NSAIDs) have been involved in various cutaneous reactions, including drug-induced rashes and the exacerbation of existing skin lesions. A study by Pandeya et al. suggests that aspirin and other NSAIDs may have a protective effect against keratinocyte cancers, indicating a complex relationship between anti-inflammatory medications and skin lesions. However, the direct impact of NSAIDs on the prevalence of SKs remains less clear, as the study primarily focused on skin cancers rather than benign lesions like SKs [39]. Rundle and Dellavalle conducted a review of the potential association between NSAIDs and non-melanoma skin cancers, noting preliminary evidence of a protective effect. However, their broader impact on skin health, particularly with regard to seborrheic keratoses, remains to be discovered in future studies [40]. This raises the question of whether NSAIDs, due to their anti-inflammatory properties, could influence the development or progression of SKs. However, further studies are needed to confirm this association.

Secondly, the role of beta-blockers in skin conditions has been documented in various studies. Aktaş et al. observed that beta-blockers might reduce the risk of certain skin cancers, potentially through their effects on angiogenesis and immune modulation. However, they also noted that some beta-blockers could be associated with an increased risk of keratotic lesions, which includes seborrheic keratoses. This finding indicates that while beta-blockers may offer a protective effect in specific contexts, on the other hand, their impact on keratinocyte proliferation or differentiation could contribute to the development of SKs in certain patient populations [41].

In our study cohort, patients with a family history of psoriasis exhibited a lower prevalence of seborrheic keratoses (SKs), suggesting that genetic predisposition to psoriasis may influence the development of these lesions. While the underlying mechanism remains unclear, it is possible that the specific inflammatory or immune profile associated with psoriasis could play a role in modulating keratinocyte proliferation and differentiation. Perez et al. proposed that genetic factors could influence the skin’s response to biologic therapies, potentially affecting the risk of developing new skin lesions. This complex interaction between psoriasis pathophysiology, genetic predisposition, and the pharmacological effects of biologic therapies may partially explain the variability in SK prevalence observed in our cohort. Further studies are needed to elucidate the mechanisms by which genetic factors related to psoriasis influence the formation of SKs [42].

An article by Gupta et al. draws attention to the development of keratotic lesions, resembling seborrheic keratoses, in psoriasis patients undergoing long-term therapy with the modified Goeckerman regimen. Our findings support their observations that SK counts are higher in patients exposed to PUVA therapy compared to those receiving UVB or systemic treatments such as methotrexate. The comparison of both datasets indicates that an extended duration of therapy has a substantial impact on the development of SKs, presumably through cumulative effects on keratinocyte behavior and skin homeostasis. Furthermore, the paper underlines the significance of the psoriatic inflammatory environment, as these lesions were exclusively present in psoriasis patients [43].

Our findings also indicate that SK counts are lower in certain psoriasis subtypes, suggesting that distinct inflammatory profiles may play a role in keratinocyte proliferation. However, larger studies are required to confirm this association and explore the underlying mechanisms [43]. In clinical practice, the appearance of new skin lesions in patients undergoing biological therapy should be evaluated carefully. While SKs are benign, distinguishing them from other skin conditions, including malignancies, is crucial. Dermatological assessment and, if necessary, biopsies can aid in establishing an accurate diagnosis and appropriate management.

## 5. Conclusions

This study provides new insights into the relationship between seborrheic keratoses (SKs), psoriasis, and biologic therapy, identifying new clinically relevant associations that warrant further investigation. A strong correlation between age and SK prevalence reinforces the well-established role of aging in SK formation, while a moderate correlation between biologic therapy duration and SK count, particularly in patients receiving TNF-alpha inhibitors, suggests that prolonged immune modulation may contribute to an increased SK burden.

An unexpected finding was the lower SK prevalence among smokers, a trend that may be linked to the immunosuppressive or vasoconstrictive effects of nicotine on keratinocyte homeostasis. In contrast, postmenopausal women and patients with osteoporosis exhibited significantly higher SK counts, suggesting a potential role of hormonal changes in SK development. Additionally, a history of PUVA therapy was associated with an increased SK burden, whereas prior UVB or methotrexate treatments appeared to have less impact, although these differences were not statistically significant.

This study has some limitations that should be acknowledged. The absence of a control group restricts our ability to establish definitive causal relationships between biologic therapies and the development of seborrheic keratoses. A matched control group of psoriasis patients not receiving biologic therapy would have strengthened our findings. However, withholding biologics from eligible patients would raise ethical concerns. Additionally, recruiting a cohort of untreated psoriasis patients for comparison would be impractical in a real-world clinical setting, as most moderate-to-severe cases are managed with systemic treatments. Instead, our analysis focused on within-group comparisons and was contextualized using the existing literature.

While these findings enhance our understanding of SK development in psoriasis patients, further prospective studies with larger, controlled cohorts are necessary to confirm these associations and explore the mechanisms underlying SK formation in this population. Clinically, these results emphasize the importance of regular dermatologic monitoring, particularly for patients undergoing long-term biologic therapy.

## Figures and Tables

**Table 1:** Duration of biologic therapy in the study group.

	n	Mean	Standard Deviation	Standard Error of the Mean	Min	Max	Median
Biologic therapy duration (months)	60	62.38	6.013	46.576	10	180	39.50

**Table 2:** Therapeutic classes of biologics in the study group.

		n	%
Therapeutic class	TNF-alpha inhibitors	18	30.0
	IL-17 inhibitors	25	41.7
	IL-23 inhibitors	17	28.3
Total		60	100.0

**Table 3:** Disease duration in the study group.

	n	Mean	Standard Deviation	Standard Error of the Mean	Min	Max	Median
Disease duration (years)	60	24.62	1.568	12.145	4	46	25.00

**Table 4:** Characteristics of disease severity scores at baseline and during follow-up.

	N	Mean	Standard Deviation	Standard Error of Mean	Min	Max	Median	Wilcoxon Test	
								Z	*p*
PASI score									
Baseline	60	26.240	1.3690	10.6045	7.1	51.3	23.850		
3 months	60	12.707	0.9802	7.5922	1.3	30.0	12.500	−6.736	<0.001
6 months	60	5.807	0.5663	4.3867	0.0	18.0	4.500	−6.593	<0.001
12 months	60	2.28	0.308	2.387	0	12	2.00	−5.969	<0.001
DLQI score									
Baseline	60	21.07	0.806	6.243	1	30	22.00		
3 months	60	9.33	0.885	6.854	0	28	9.00	−6.628	<0.001
6 months	60	4.35	0.572	4.433	0	23	3.00	−5.991	<0.001
12 months	60	2.00	0.385	2.986	0	18	1.00	−5.367	<0.001

**Table 5:** Overview of comorbidities in the study cohort.

	PRESENT		ABSENT	
	n	%	n	%
Biologic inflammatory syndrome	28	46.7	32	53.3
Endocrine dysfunction	3	5.0	57	95.0
Diabetes	11	18.3	49	81.7
Urinary tract infection history	7	11.7	53	88.3
Cardiovascular disease	22	36.7	38	63.3
Musculoskeletal disorders	14	23.3	46	76.7
Asthma	2	3.3	58	96.7
Chronic obstructive pulmonary disease	9	15.0	51	85.0
History of ischemic heart disease or stroke	2	3.3	58	96.7
History of surgical procedures	9	15.0	51	85.0
Renal impairment	2	3.3	58	96.7
Neurological disorders	1	1.7	59	98.3
Hepatic disease	35	58.3	25	41.7
Anemia	5	8.3	55	91.7
Thrombocytopenia	4	6.7	56	93.3
Total			60	100.0

**Table 6:** Analysis of the count of seborrheic keratoses based on patients’ demographic characteristics.

Number of SKs		N	Mean	Std Deviation	Standard Error of Mean	Min	Max	Median	Mann–Whitney Test
**Gender**	Male	40	6.35	4.544	0.718	0	23	6.00	U = 382.500
	Female	20	5.70	2.940	0.657	0	11	5.50	*p* = 0.783
Environment	Urban	42	5.67	4.177	0.645	0	23	6.00	U = 291.000
	Rural	18	7.22	3.671	0.865	3	16	7.00	*p* = 0.158

**Table 7:** Analysis of the number of existing seborrheic keratoses based on the clinical characteristics of patients, previous medication, and disease progression.

Number of Existing Seborrheic Keratoses		N	Mean	Standard Deviation	Standard Error of Mean	Min	Max	Median	Mann–Whitney/Kruskal–Wallis Test
**Smoking status**	Smoker	26	5.23	4.033	0.791	0	16	5.00	U = 305.500
	Non-smoker	34	6.82	4.011	0.688	0	23	7.00	*p* = 0.041
Alcohol consumption	Occasional	36	6.08	3.752	0.625	0	16	6.00	U = 414.500
	Does not consume	24	6.21	4.578	0.934	0	23	5.50	*p* = 0.791
Menopause	Yes	16	6.31	2.676	0.669	2	11	6.50	U = 13.500
	No	4	3.25	2.986	1.493	0	7	3.00	*p* = 0.080
Osteoporosis	Yes	8	9.63	5.680	2.008	5	23	7.50	U = 96.000
	No	52	5.60	3.533	0.490	0	16	5.50	*p* = 0.014
Chronic UV exposure	Occasional	20	6.55	3.953	0.884	2	16	6.00	U = 372.500
	Absent	40	5.93	4.153	0.657	0	23	6.00	*p* = 0.665
History of sunburn	Yes	16	6.00	3.464	0.866	2	14	5.00	U = 336.000
	No	44	6.18	4.299	0.648	0	23	6.00	*p* = 0.788
History of keratoacanthoma	Yes	3	15.33	8.021	4.631	7	23	16.00	U = 18.000
	No	57	5.65	3.215	0.426	0	14	6.00	*p* = 0.016
Phototype	II	19	5.42	3.339	0.766	0	11	6.00	H = 2.550
	III	38	6.71	4.380	0.711	0	23	6.00	*p* = 0.279
	IV	3	3.33	3.055	1.764	0	6	4.00	
Biologic-naïve patient	Yes	47	6.11	4.340	0.633	0	23	6.00	U = 267.500
	No	13	6.23	3.004	0.833	0	12	6.00	*p* = 0.493
Biological therapy class	TNF-alpha inhibitors	18	8.50	4.866	1.147	3	23	7.50	H = 8.254
	IL-17 inhibitors	25	5.60	3.452	0.690	0	12	5.00	*p* = 0.016
	Il-23 inhibitors	17	4.41	2.830	0.686	0	9	5.00	
History of beta-blocker use	Yes	14	7.50	3.777	1.010	1	16	7.00	U = 211.000
	No	46	5.72	4.097	0.604	0	23	5.00	*p* = 0.051
History of NSAIDs use	Yes	25	6.84	3.859	0.772	0	16	7.00	U = 345.000
	No	35	5.63	4.187	0.708	0	23	5.00	*p* = 0.163
Family history of psoriasis	Yes	15	4.40	3.112	0.804	0	11	4.00	U = 212.000
	No	45	6.71	4.208	0.627	0	23	6.00	*p* = 0.031
Psoriatic arthropathy	Absent	34	6.12	4.578	0.785	0	23	6.00	H = 1.194
	Axial	7	6.43	3.599	1.360	0	11	7.00	*p* = 0.755
	Peripheral	10	6.50	3.171	1.003	2	12	6.00	
	Mixed	9	5.56	3.712	1.237	2	14	4.00	
Genital involvement	Yes	5	5.00	2.449	1.095	2	8	4.00	U = 114.000
	No	55	6.24	4.181	0.564	0	23	6.00	*p* = 0.550
Scalp involvement	Yes	50	6.28	4.276	0.605	0	23	6.00	U = 239.500
	No	10	5.40	2.836	0.897	0	9	6.50	*p* = 0.834
Palmoplantar involvement	Yes	23	5.70	2.566	0.535	2	12	5.00	U = 392.500
	No	37	6.41	4.781	0.786	0	23	6.00	*p* = 0.614
Nail involvement	Yes	50	6.00	3.156	0.446	0	16	6.00	U = 241.500
	No	10	6.80	7.315	2.313	0	23	5.50	*p* = 0.865

**Table 8:** Correlation between seborrheic keratosis count and other quantitative variables in the cohort.

Number of Seborrheic Keratoses Correlated with	Pearson Correlation Coefficient (r)	*p* Value		
			95% CI (Confidence Interval)	Interpretation
**Age**	**0.717**	<0.001	0.566 ÷ 0.821	Strong, direct proportional correlation, statistically significant (SS)
BMI	0.160	0.221	−0.097 ÷ 0.398	Weak, direct proportional correlation, not significant (NS)
Biologic therapy duration (months)	0.270	0.037	0.017 ÷ 0.490	Weak-to-moderate direct proportional correlation, SS
Psoriasis onset (years)	0.122	0.353	−0.136 ÷ 0.365	Weak, direct proportional correlation, NS
Baseline PASI score	0.121	0.358	−0.137 ÷ 0.364	Weak, direct proportional correlation, NS
Baseline DLQI score	−0.083	0.531	−0.330 ÷ 0.175	No correlation, NS

## Data Availability

No new data were created or analyzed in this study. Data sharing is not applicable to this article.

## Abbreviations

The following abbreviations are used in this manuscript:

PASI	Psoriasis Area and Severity Index
DLQI	Dermatology Life Quality Index
MTX	Methotrexate
PUVA	Psoralen and ultraviolet A therapy
UVB	Ultraviolet B therapy
NSAID	Non-steroidal anti-inflammatory drug
SK	Seborrheic keratosis
IL	Interleukin
TNF	Tumor necrosis factor
COPD	Chronic obstructive pulmonary disease
RCM	Reflectance confocal microscopy
